# A Comprehensive Review Emphasizing Anatomy, Etiology, Diagnosis, and Treatment of Male Urethral Stricture Disease

**DOI:** 10.1155/2019/9046430

**Published:** 2019-04-18

**Authors:** Wesley Verla, Willem Oosterlinck, Anne-Françoise Spinoit, Marjan Waterloos

**Affiliations:** ^1^Ghent University, Faculty of Medicine and Health Sciences, Ghent, Belgium; ^2^Department of Urology, Algemeen Ziekenhuis Maria Middelares, Ghent, Belgium

## Abstract

To date, urethral stricture disease in men, though relatively common, represents an often poorly managed condition. Therefore, this article is dedicated to encompassing the currently existing data upon anatomy, etiology, symptoms, diagnosis, and treatment of the disease, based on more than 40 years of experience at a tertiary referral center and a PubMed literature review enclosing publications until September 2018.

## 1. Introduction

Urethral stricture disease can develop throughout the entire length of the male urethra and can be caused by a large variety of etiologies. Also, urethral strictures give rise to a wide range of symptoms and warrant a specific diagnostic work-up before proceeding to any treatment modality.

The management of urethral stricture disease has a profound history and may embody one of the oldest documented urological entities known to mankind. In the second half of the 20^th^ century, urologists have attempted to find solutions to treat both simple and complex urethral strictures and, over the last few decades, research has mainly been focused on refining the existing procedures to mitigate the negative postoperative consequences. However, despite the substantial scientific progress on this subject, a numerous amount of studies has revealed the insufficient knowledge about urethral stricture surgery among urologists and has shown that patients with urethral strictures are generally offered an inadequate treatment option [1–3].

Against this background, the article outlines the existing data about anatomy, etiology, symptoms, diagnosis, and treatment of male urethral stricture disease.

## 2. Anatomy

**Figure 1:**
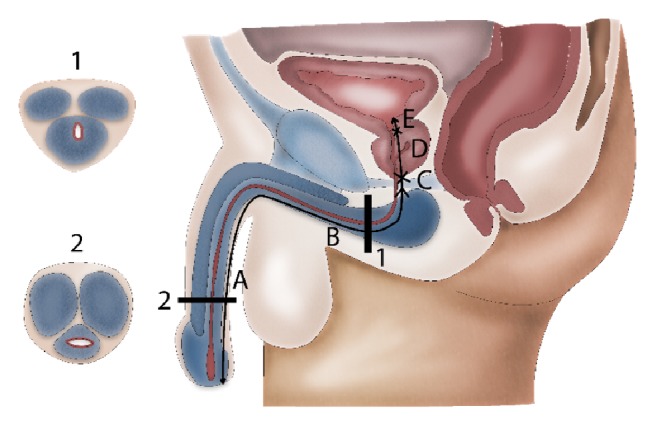
Anatomy of the male urethra.* 1 = bulbar urethra (urethra runs dorsally through corpus spongiosum); 2 = penile urethra (urethra runs centrally through corpus spongiosum). A = penile urethra; B = bulbar urethra; C = membranous urethra; D = prostatic urethra; E = bladder neck*.

The terminology describing male urethral anatomy is often used incorrectly and thus needs clarification. In fact, the male urethra consists of the following segments (from bladder neck to meatus urethrae): the posterior urethra, containing the prostatic urethra and the membranous urethra, and the anterior or spongious urethra (embedded in corpus spongiosum), containing the bulbar urethra (between the membranous urethra and the penoscrotal angle) and the penile urethra (between the penoscrotal angle and the meatus urethrae) (Figure 1). Diseases of the prostatic urethra are beyond the scope of this article as they largely overlap with specific prostate diseases.

## 3. Etiology

The etiology of urethral stricture disease mainly involves the following: idiopathic, iatrogenic, external trauma, infection, and lichen sclerosus. In 2013, a comparative analysis showed that urethral strictures in India are proportionally more caused by an external trauma and less by an iatrogenic cause, when compared to the USA and Italy [4]. Meanwhile, in the Western World, the most important stricture etiology is iatrogenic [4–6] and developing countries primarily face infectious strictures after venereal infections or after a nonspecific urethritis [7]. As regards lichen sclerosus, a skin condition with an important predilection for the anogenital region, its urethral involvement is a well-known aspect of the disease and potentially gives rise to urethral strictures at the penile or bulbar site [8, 9]. Furthermore, it must be underlined that a substantial amount of stricture etiologies remains unknown, even after thorough evaluation of the patient's history.

### 3.1. Iatrogenic Stricture Etiology

The medical world is responsible for a substantial amount of urethral strictures [5]. Every transurethral intervention (e.g., catheter insertion, introduction of surgical instruments, etc.) can damage the urethral mucosa and lead to subsequent stricture formation, even if performed adequately [5]. Therefore, all medical practitioners should keep this in mind and carefully consider their indications before proceeding to any transurethral manipulation. Failed hypospadias repair represents another important iatrogenic cause of strictures, especially in younger patients [5]. In a relatively older patient population, local prostate cancer treatments, mainly involving radical prostatectomy or radiation therapy, are an upcoming etiology and bring along strictures that are very challenging to treat [5]. Less frequently, strictures are due to ischemia of the corpus spongiosum which may occur after hypothermia or extracorporeal circulation, for instance, during cardiac or neurosurgery [5]. In these cases, strictures typically involve the complete anterior urethra because this entire segment strongly relies on the spongious blood supply.

### 3.2. External Trauma

External trauma leading to a pelvic fracture specifically threatens the membranous part of the male urethra either by a shear injury resulting from the movement of the pelvic bones or by a laceration injury caused by bony fragments cutting into the urethra. These phenomena may result in a partial or total rupture of the urethra. The associated hematoma formation then further separates both urethral ends and causes such a disruption defect in between.

Straddle injuries or a trauma directly impacting the perineum may damage the bulbar urethra as this part of the urethra gets crushed between the area of impact and the pubic bone. Generally, these injuries lead to stricture formation at the site of urethral damage and are usually accompanied by an important perineal hematoma due to an associated rupture in the surrounding spongious tissue.

In case of severe penile fractures, the rupture of the cavernous bodies can be associated with a rupture of the penile urethra which may lead to subsequent stricture formation.

## 4. Symptoms and Diagnosis

### 4.1. Patient History

Patients with a urethral stricture mostly complain about obstructive voiding symptoms. The most apparent symptom is weakening of the urinary stream. However, it is important to understand that all degrees of obstructive voiding can be present, ranging from a perfectly normal urinary stream to urinary retention. In case of a discrete urethral stricture and/or a slowly progressive onset of symptoms, the patient can indeed report a total absence of obstructive voiding symptoms as the detrusor muscle may compensate the lower urinary tract obstruction by hypertrophy. Other obstructive voiding symptoms may involve hesitancy, intermittency, straining, post-void dribbling, incomplete emptying of the bladder, and spraying (especially in meatal strictures). Apart from these, development of an overactive bladder is frequent as well and brings along complaints of urgency and frequency.

Other complaints such as hematuria or pollakisuria are also possible, although they are likely to be the result of a stricture related complication such as urinary stones, urethritis, or an infection of the prostate, epididymis, or testicle.

The presence of a urethral stricture should always be suspected in case of repetitive infections of the prostate, epididymis, or testicle.

Next to symptom assessment, history taking should focus on stricture etiology, previous interventions, relevant medical history and comorbidities.

### 4.2. Physical Examination

During physical examination, the clinician should palpate the urethra to identify fibrotic tissue and look for skin changes (e.g., lichen sclerosus), the presence of cellulitis, fistulas, or abscesses, the presence and quality of foreskin to potentially use in later urethral reconstruction and the presence of scars from prior surgery. These surgical scars may reveal important information about the type of prior reconstruction which is sometimes unknown to the patient.

Ideally, the examination of the patient also includes a digital rectal examination (benign prostatic hyperplasia; prostatitis) and an evaluation of the external genitals which might reveal (the consequences of) an epididymitis or an orchitis.

Be that as it may, imaging studies remain essential to evaluate the entire male urethra as only part of it can be evaluated by physical examination.

### 4.3. Technical Investigations

#### 4.3.1. Uroflowmetry

The maximal urinary flow rate (Qmax) of an adult man with a healthy lower urinary tract is estimated to be >15 mL/s [10]. A Qmax of <15 mL/s is considered suspicious for lower urinary tract obstruction and requires further diagnostic evaluation. Apart from Qmax, it is also important to interpret the shape of the flow curve as in patients with a urethral stricture, uroflowmetry may typically reveal a curve with a plateau-shape at the level of the Qmax [11–13].

It must be underlined that uroflowmetry studies with a voided volume < 150 mL can lead to a less valuable interpretation [12].

#### 4.3.2. Urethroscopy

Urethroscopy is a fast and relatively easy way to diagnose a urethral stricture. This investigation provides information about the location and the remaining caliber of the narrowed urethra (to pass or not to pass with the cystoscope). If the stricture is too narrow to allow passage of the cystoscope, no further information about the proximal urethra can be obtained with this diagnostic modality. In these cases it can be helpful to introduce a smaller caliber ureteroscope (4.5 or 6 Fr) which is able to pass through the strictured area and which thus provides further proximal information [14].  Apart from that, urethroscopy is also unable to provide any information about the surrounding spongiofibrosis. Given these drawbacks, urethroscopy alone is often considered insufficient for a thorough diagnostic work-up and additional imaging studies are mostly warranted.

#### 4.3.3. Urethrography

**Figure 2:**
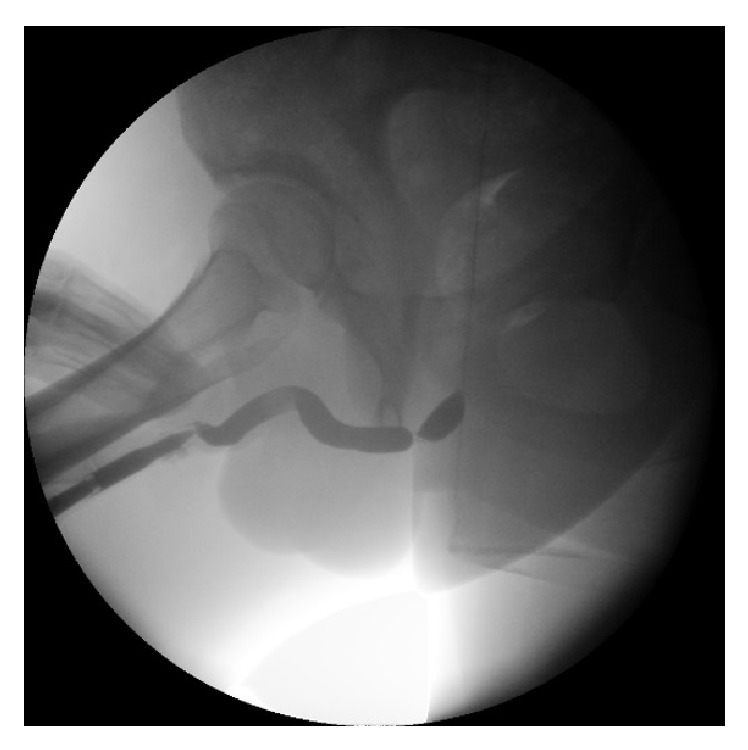
Retrograde urethrography.* Retrograde urethrography shows an isolated, short, bulbar urethral stricture.*

A retrograde urethrography (RUG), in which contrast is injected through the urethral meatus, is capable of visualizing the entire urethra (except in cases with a total obliteration of the urethral lumen) up to the sphincter and even up to the bladder if patients can relax the sphincter enough to allow passage of contrast through the prostatic urethra and bladder neck (Figure 2). However, a RUG alone often results in insufficient distension of the urethra proximal to the stricture which may lead to incomplete information about the proximal stricture extent and the condition of the more proximal urethra. In this case, an additional voiding cysto-urethrography (VCUG) after filling the bladder with contrast (either after RUG or by instillation through a suprapubic catheter) may solve this problem and address the need for additional information.

The combination of both RUG and VCUG provides a comprehensive image of the entire urethra and reveals valuable information about the number of strictures, stricture length, stricture location, and the remaining caliber of the narrowed urethra. Nonetheless, RUG and VCUG studies require careful interpretation and several drawbacks must be kept in mind. A prestenotic dilation, for instance, can mask the presence of a urethral stricture or interfere with the observed stricture length, especially in the bulbar urethra. In these cases, an additional image in profile view can be very useful. Furthermore, estimated stricture length, particularly at the bulbar site, should always be interpreted carefully as this result poorly correlates with the intraoperatively measured stricture length [15]. This can be explained by the fact that a 3-dimensional situation is projected on a 2-dimensional image and by the fact that the observed stricture length importantly depends on the patient's positioning and the provided penile traction during the investigation [15]. RUG and VCUG are further limited by the fact that—similar to urethroscopy—none of these studies provide any information about the surrounding spongiofibrosis.

#### 4.3.4. Urethral Ultrasound

**Figure 3:**
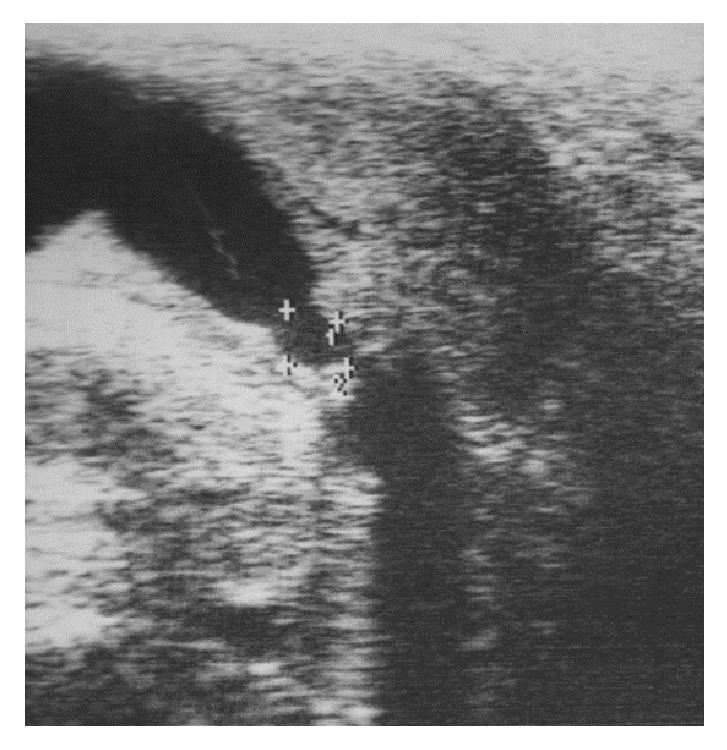
Ultrasound of the bulbar urethra.* Ultrasound shows an irregular, hyperechogenic zone in the bulbar urethra, representing a bulbar urethral stricture*.

A urethral ultrasound may be useful in the diagnostic work-up of urethral strictures, particularly because it measures stricture length more adequately and because it reveals information about the surrounding spongiofibrosis [15]. During this investigation, a linear 7,5 MHz probe is placed sagitally against the region of interest: ventral penis for penile strictures, perineal for bulbar strictures. The urethra is then visualized as a hypoechogenic band with an 8 to 10 mm diameter (after instillation of a physiologic solution through a Foley catheter at the level of the meatus) and urethral strictures are represented as thick, irregular, and hyperechogenic zones in and around the depicted urethra (Figure 3).

Despite the aforementioned assets of urethral ultrasound, this imaging study is vastly underused in clinical practice and urethrography remains the routine diagnostic modality, principally because its rapid information is sufficient for the reconstructive urologist. Furthermore, as a urethral ultrasound takes longer to perform, there is a prolonged period of retrograde injection of a physiologic solution which is uncomfortable for the patient when awake.

#### 4.3.5. Magnetic Resonance Imaging (MRI) and Computed Tomography (CT) Scan

MRI is a very useful tool in case of tumor related urethral stricture disease. This imaging study can adequately demonstrate the extent of the disease in the surrounding tissues, for instance, into the cavernous bodies, which is of utmost importance for the subsequent surgical procedure. Further routine use in clinical practice is rather debatable. Nonetheless, Oh et al. advocate for MRI in case of complex trauma leading to a completely obliterated posterior urethra [16] and, more recently, Joshi et al. described a novel MRI protocol which leads to a reliable measurement of the urethral gap after pelvic fracture related urethral injuries [17]. This novel MRI technique could be very useful to plan and guide subsequent urethral reconstruction in these often complex cases [17].

A CT voiding urethrography can provide useful information in case of stricture related fistulas [18].

## 5. Treatment

### 5.1. Dilations

Urethral dilation represents one of the oldest urological procedures known to mankind. Throughout history, urologists have invented all sorts of instruments to progressively dilate urethral strictures up to a normal caliber. However, since the development of direct vision internal urethrotomy (DVIU), urethral dilation as a primary treatment for urethral stricture disease has decreased and research has started focusing on repetitive (auto)dilations as a strategy to prevent or delay stricture recurrence after DVIU (cf. infra) [19, 20].

As the mechanism of urethral dilation implies rupturing the urethral mucosa at the least scarred region of the stricture, it allows subsequent urine diffusion in the created defect and peri-urethral tissues which further nourishes the formation of scar tissue. Hence, a high stricture recurrence rate can be expected, either in the short or in the long term. Accordingly, durable success rates after urethral dilation for a primary, short urethral stricture lie between 50 and 60% but decrease to about 20% for strictures longer than 2.0 cm [21, 22]. Nonetheless, these results need to be put in perspective as DVIU can also cause urinary extravasation into the peri-urethral tissues and since Steenkamp et al. described no statistically significant difference in surgical outcome between DVIU and urethral dilation [21].

Potential risks that are associated with single or repetitive (auto)dilations include urethral hemorrhage, urinary tract infection, and sepsis. Furthermore, it is advised not to dilate the urethra in case of a present urinary tract infection.

### 5.2. Endoscopic Treatment

#### 5.2.1. Direct Vision Internal Urethrotomy (DVIU)

DVIU represents the basis of endoscopically treating urethral strictures and is inspired by a French idea, born in the 19^th^ century (Civiale, 1817; Maisonneuve, 1848). This treatment principally differs from urethral dilation as it involves an intervention which is guided by the direct vision of the surgeon. During this procedure, a longitudinal incision is made over the entire stricture length into healthy urethral tissue, after which the gap between the wound edges is expected to be re-epithelialized.

Today, many urologists feel comfortable treating urethral strictures with endoscopic urethrotomy. Most likely, this popularity is mainly a result of its short learning curve, its relatively fast and simple character, and its paucity of major complications, rather than its intrinsic surgical outcome. Moreover, in many urology practices, a thorough knowledge of and/or experience with open urethral reconstruction are/is lacking [23].

*(1) Indications. *The sole indication for DVIU is a primary, isolated, short (<1.5 cm), bulbar urethral stricture. This recommendation is based upon several negative prognostic factors impeding success rates of DVIU: number of previous urethrotomies (and stricture-free interval hereafter), stricture length, number of strictures, stricture location, and the amount of surrounding spongiofibrosis [24, 25].

*(2) Results. *DVIU for primary urethral strictures <1.5 cm entails the best surgical outcome, with success rates ranging up to 80% in case of strictures <1 cm [26]. When performing DVIU for longer strictures, success rates drop to about 20% [26]. Moreover, Steenkamp et al. have shown that every additional centimeter to be treated with DVIU brings along an extra risk factor (RR: 1.22) for stricture recurrence [21].

*(3) Therapeutic Options to Prevent or Delay Stricture Recurrence after Urethrotomy*

*Repetitive (Auto)Dilation*. Repetitive (auto)dilation is frequently administered as an adjuvant therapy after DVIU to prevent or delay stricture recurrence. This strategy has been evaluated by several retrospective series which have shown a limited benefit over DVIU only and these findings have been corroborated by a recent systematic review and meta-analysis [27–30]. These studies, however, provide no data about which specific patient groups might benefit more or less from these adjuvant dilations and it has been described that repetitive (auto)dilations are associated with a poor quality of life [31].

*Injection or Instillation with Corticosteroids. *Post-urethrotomy injection or instillation with corticosteroids has been evaluated by two randomized [32, 33] and two nonrandomized [34, 35] series, three of which have shown a clear benefit over DVIU only in strictures <2 cm [32, 34, 35]. However, all of these reports are based on a poor-quality study design and thus no clear conclusions can be drawn about the true value of corticosteroids in this setting.

*Injection with Low-Dose Mitomycin C (MMC). *Low-dose MMC (0.1 mg in 2 mL, 5%) injections in the freshly incised urethral stricture have been described with beneficial results in an initial small randomized, controlled trial (n=20) [36]. The rationale behind this strategy lies within the antifibrotic and anticollagen properties of the MMC substance. More recent studies, involving a large randomized, controlled trial [37] and a descriptive study in 37 patients [38], have confirmed the benefit of adding these injections over DVIU only.

Nevertheless, the use of post-urethrotomy MMC injections has not exactly found its way into routine clinical practice. The experienced serious adverse events following extravasation of MMC after intravesical instillations, even though these concentrations of MMC are 20 times higher, might be an explanation for this. Furthermore, this trend may be further encouraged by the presence of all valuable alternatives, as mentioned above.

#### 5.2.2. Metallic Endoluminal Stents

Metallic endoluminal stents are endoscopically inserted after incision of the urethral stricture and are manufactured to maintain a sufficient caliber at the level of the diseased urethra. These stents may be particularly interesting in short urethral strictures as they allow re-epithelization from both extremities. In more extensive stricture disease, however, the formation of granulation tissue may overgrow the meshes of the inserted stent and thus lead to a partial or complete obliteration of the stent's endolumen.

These endoluminal stents were enthusiastically introduced in the 1990s, especially for short, recurrent bulbar strictures as they offered promising short-term success rates [39]. However, in the long-term, devastating problems occurred in patients after endoluminal stent implantation including restenosis due to overgrowth, stent migration, encrustation, and infection [39]. Thereafter, endoluminal stents rapidly decreased in popularity and several reconstructive techniques were advocated for urethroplasty after endoluminal stent failure [39].

#### 5.2.3. Laser Evaporation of the Stricture

The laser technique is capable of evaporating the entire urethral stricture, but it destroys the epithelium of the urethra at the same time. Considering this, holmium laser urethrotomy is specifically indicated for short urethral strictures, because, in these cases, re-epithelization may be expected even sooner than in classic urethrotomy. Moreover, there is limited evidence that, for these strictures, the recurrence rates within 1 year of follow-up are significantly lower after laser urethrotomy, when compared to cold-knife incision [40]. In case of longer urethral strictures, less favorable results are to be expected from this treatment modality, particularly when the urethral epithelium has been disintegrated over the entire length of the treated stricture. However, these auspicious results should be put in perspective as they are supported by a limited amount of studies, with small sample size, short follow-up and poor description of the study design and methods [40].

### 5.3. Open Reconstructive Treatment

Urethroplasty, the open reconstructive treatment for urethral strictures, is associated with significantly better long-term success rates than dilation or any endoscopic treatment option [26]. Over time, a tremendous amount of surgical techniques has been described and gradually refined, providing a very rich armamentarium for the reconstructive urologist. The exact choice of technique for a particular patient with a particular case of urethral stricture disease will depend on numerous factors, at least including the following: previous urethral treatments, number of strictures, stricture length, stricture location, stricture etiology, comorbidities and the quality of the corpus spongiosum, the surrounding tissues, and potential graft sites. Hence, a thorough diagnostic work-up is indispensable and of utmost importance when selecting the most adequate treatment option.

#### 5.3.1. Timing of Surgery

Urethroplasty should be timed adequately and needs to take place after full maturation of the stricture. Following this logic, the authors believe that postponing urethral reconstruction until 3 months after the latest transurethral manipulation is the ideal approach, although there is no true evidence to support this specific statement. The rationale behind our timing is that the introduction of even a small caliber instrument may rupture the strictured area, causing a significant problem in the intra-operative determination of the distal extent of the stricture as a transurethral catheter or Beniqué might fluently pass through the dilated, but diseased urethra. This in turn could lead to an insufficient urethroplasty procedure, leaving fibrotic tissue, and thus stricture disease, behind.

Considering this, urinary diversion will need to be guaranteed by placing a suprapubic catheter in case of acute urinary retention.

#### 5.3.2. Preoperative Work-Up

The key-point in preoperative work-up is to assure that the patient's urine is sterile during urethroplasty as a urinary tract infection can complicate the postoperative course and might contribute to urethroplasty failure. Therefore, it is advised to perform a urinalysis with urine culture and antibiogram one week preoperatively and to start with appropriate antibiotics 24 hours before surgery. This is especially important in patients with a suprapubic catheter, in whom the risk of contaminated urine is substantially higher. In case of a sterile urine portion, a single dose of cefazoline or a quinolone is administered at the start of the operation. In these cases, it is unnecessary to routinely perpetuate the antibiotic treatment regimen after any urethroplasty since this would only endorse the increasing problem of resistant microorganisms.

#### 5.3.3. Urethral Access

**Figure 4:**
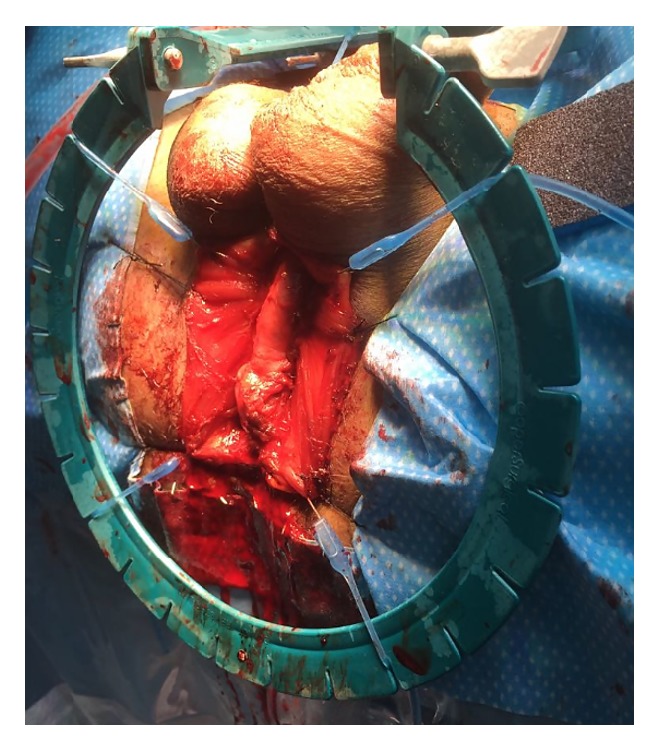
Exposure of the bulbar urethra.* The bulbar urethra is exposed using 4 stay sutures and a Lone Star retractor*.

For a long period of time, the authors have been using a midline perineal skin incision to access the bulbar and posterior urethra. This incision gives an excellent exposure with a minimal risk of wound dehiscence after closure and it has the advantage to be less painful than an inverted-U or *λ* incision [41]. After the skin incision, the subcutaneous fat tissue is further dissected until the level of the bulbospongiosus muscle, which is then incised longitudinally on the midline and separated from the corpus spongiosum. Further exposure can be obtained by fixating the muscle to the perineal skin with 4 stay sutures and by applying a self-retaining retractor with multiple stay hooks (Figure 4).

**Figure 5:**
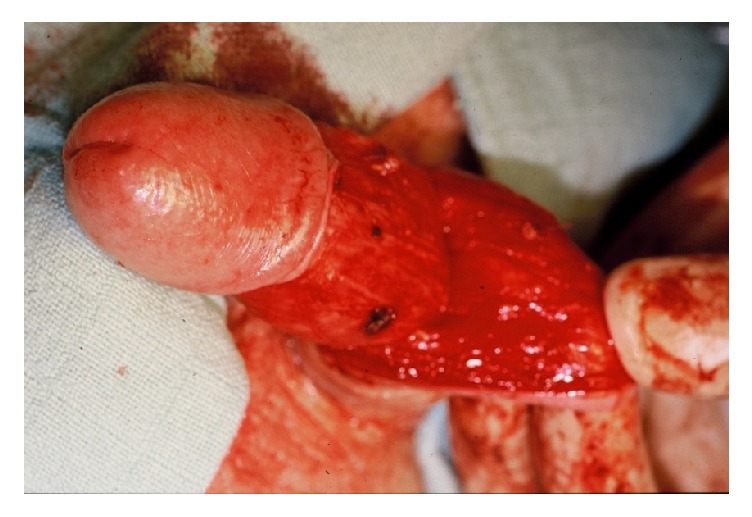
Exposure of the penile urethra.* After a circumferential incision, the penis is degloved along Buck's fascia providing exposure of the entire penile urethra*.

To access the penile urethra, a circumferential skin incision about 0.5 cm below the glans is an excellent approach (Figure 5). This incision will provide an excellent, well vascularized coverage of the reconstructed area and minimizes the risk of postoperative fistulation. After this skin incision, the penis can be degloved along Buck's fascia, following the virtually avascular plane in between, which results in an easy exposure of the entire penile urethra [42, 43].

An alternative approach to access the penile urethra was described by Kulkarni et al. and involves invagination of the penis through a perineal skin incision, thus accessing the entire penile urethra without the need for a penile skin incision [44]. This approach offers a perfect coverage of the penile site of reconstruction and allows total anterior urethral reconstruction through one perineal incision.

#### 5.3.4. Surgical Techniques

**Figure 6:**
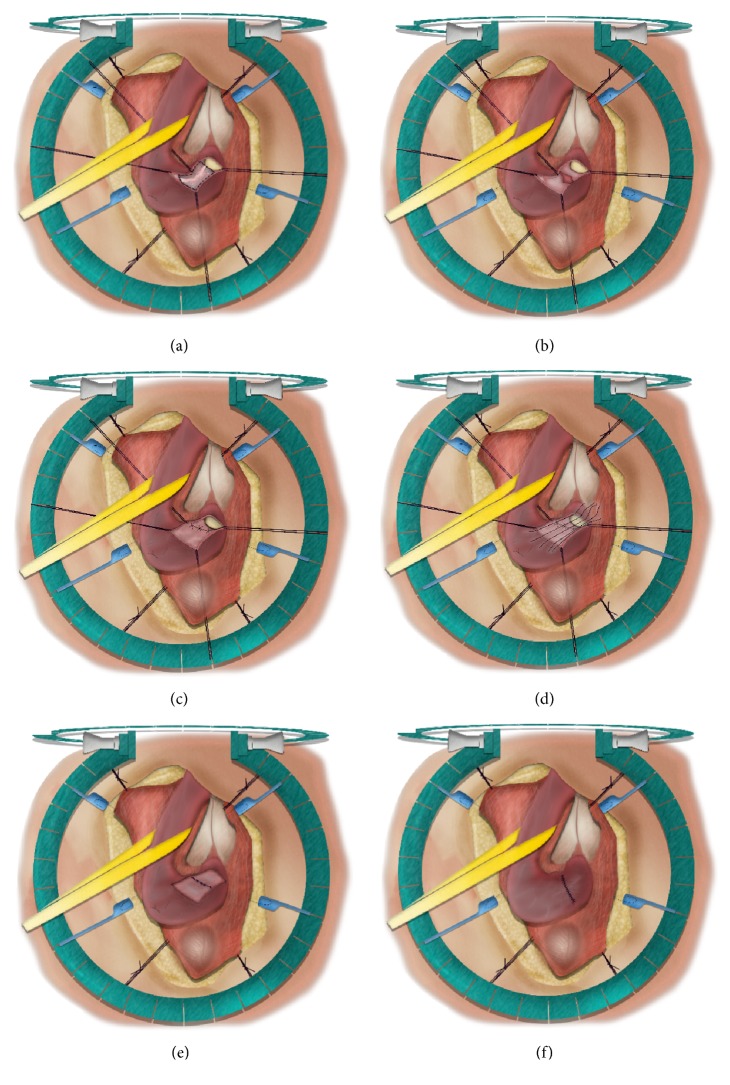
Nontransecting end-to-end urethroplasty.* (a) = excision of the stricture and surrounding spongiofibrosis after dorsal, longitudinal incision; (b) = ventral spatulation of the proximal and distal urethral end; (c) = transverse closure of the ventral urethral plate; (d, e) = transverse closure of the dorsal urethra; (f) = spongioplasty.*

*(1) End-to-End Urethroplasty. *End-to-end urethroplasty or excision and primary anastomosis (EPA) urethroplasty represents the surgical technique with the best long-term surgical success, entailing a composite success rate of 93.8% [45, 46]. During this procedure, the diseased segment of the urethra is excised and replaced by adjacent healthy urethral tissue, without the need for grafts or flaps to bridge the gap (Figure 6) [47].

Be that as it may, the indications for this surgical technique are restricted by the limited elasticity of the bulbar urethra. In fact, only strictures up to 3.0 cm are to be treated with end-to-end urethroplasty because the excision of longer segments would hamper the creation of a well vascularized and tension-free anastomosis, which is crucial for a successful procedure. The intrinsic elasticity of the bulbar urethra is estimated to be around 25% and, assuming an average bulbar urethral length of 10 cm, a gap of up to 2.5 cm can be bridged. Further length can be gained progressively by performing additional maneuvers involving extensive proximal and distal urethral mobilization, cleavage of the cavernous bodies, supracrural rerouting, and inferior pubectomy [48]. If, even after these length-gaining maneuvers, the gap between both urethral ends remains too large, an augmentation using free grafts or pedicled flaps will be inevitable [47].

Recommendations regarding urethroplasty of the bulbar urethra are currently highly inconsistent and controversial opinions are reported in literature: the International Consultation on Urologic Diseases (ICUD) advises end-to-end urethroplasty as the technique of first choice in all isolated, short (≤ 3.0 cm), bulbar urethral strictures while others may argue that it is only indicated after bulbar trauma [48–50]. Apart from that, end-to-end urethroplasty is also recommended for posterior urethral strictures after pelvic fractures or after prostate surgery or radiation [47, 51–53]. Herein, the scar tissue is entirely excised and a bulbomembranous or bulboprostatic anastomosis is created. A cystoscope or a curved sound may be introduced in the suprapubic tract, down to the proximal urethral portion, to guide the surgeon in his/her surgical dissection and creation of the anastomosis [47, 51]. In very unusual circumstances, a combined abdominoperineal approach might be necessary [51]. In most of these patients with posterior urethral strictures, the elaborated anastomotic repair technique defined by Webster et al. will suffice to restore urethral continuity [48]. If this would not be the case, substitution or staged urethroplasty remains a viable option.

End-to-end urethroplasty is contraindicated in most penile urethral strictures, even in short ones, as EPA in this segment may lead to penile shortening and chordee.

*Nontransecting End-to-End Urethroplasty. *Traditionally, end-to-end urethroplasty included full thickness transection of the corpus spongiosum at the level of the stricture. However, as EPA only requires the excision of the narrowed segment and the surrounding spongiofibrosis, a full thickness transection of the corpus spongiosum, with the bulbar/urethral arteries within it, is usually unnecessary [47]. Against this background, Jordan et al. introduced the idea of a “nontransecting” or “vessel-sparing” technique in 2007 [54] which was later slightly modified and popularized by Andrich and Mundy (Figure 6) [55]. Many urethroplasty centers have adopted this technique ever since and promising results—in line with the success rates of the transecting technique—have been reported [47, 56–59]. This nontransecting technique aims to reduce surgical trauma, especially to the bulbar/urethral arteries embedded in the corpus spongiosum. Preserving these arteries potentially reduces the risk of postoperative erectile dysfunction or glans ischaemia. Apart from that, vessel-sparing could also be beneficial for subsequent urethral interventions requiring a well sustained vascular milieu, such as free graft urethroplasty or the implantation of an artificial urinary sphincter [47]. So far, these potential benefits are only assumptions as there is only one retrospective series suggesting a functional benefit for nontransecting EPA over transecting EPA [59]. Prospective randomized, controlled trials with validated questionnaires will be needed to corroborate these suggestions.

This nontransecting variant of end-to-end urethroplasty has also been introduced to treat posterior urethral strictures [60, 61]. However, in these strictures, the bulbar arteries and the cavernous vasculonervous bundles may already be obliterated or damaged due to the pelvic fracture or previous prostate treatments, abolishing the potential benefits of vessel preservation. Furthermore, the anatomical proximity of the membranous urethra to the urinary sphincter and the cavernous vasculonervous bundles should be taken into account and, if possible, a sphincter-sparing variant of nontransecting end-to-end urethroplasty may be superior in terms of continence preservation [62].

**Figure 7:**
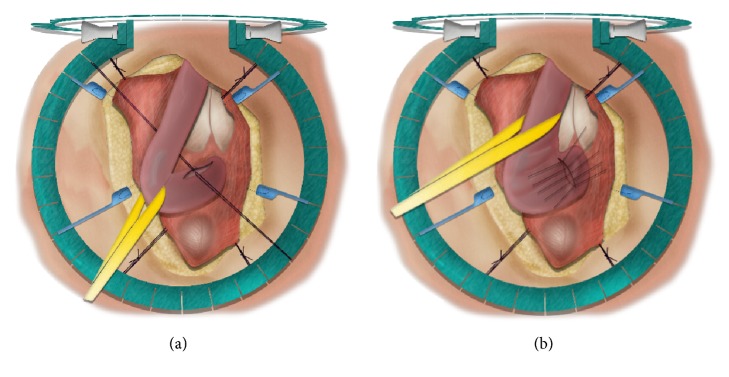
Heineke-Mikulicz stricturoplasty.* (a) = longitudinal incision over the stricture; (b) = transverse closure of the incision.*

For short (<1.0 cm) and not too narrow strictures, a Heineke-Mikulicz urethroplasty can be performed with excellent success rates (Figure 7) [63]. In this subtype of nontransecting procedure, the stricture is longitudinally incised and then closed transversely without excising the fibrotic tissue. Some call this technique a stricturoplasty, rather than a true urethroplasty.

*(2) Free Graft Urethroplasty. *From the moment a stricture is no longer an indication for end-to-end urethroplasty, a substitution urethroplasty is unavoidable [64]. Herein, the use of a free graft represents the easiest and most straightforward technique to treat strictures from the meatus urethrae up to the posterior urethra. These free grafts can be harvested from several sites such as the preputium, the penile shaft, the oral cavity (buccal or lingual mucosa), the tunica vaginalis, and exceptionally the bladder mucosa [64]. Various manners have been described to suture the harvested graft onto or into the opened urethra (ventral onlay, dorsal onlay, dorsolateral onlay, dorsal inlay, and combinations), but tubularized grafts are to be avoided since these results are far less favorable than the aforementioned substitution techniques [64]. Furthermore, it is well known that free graft procedures provide worse outcomes at the penile urethra than at the bulbar urethra [64]. Most likely, this is the result of a more limited amount of corpus spongiosum at the penile site and its proximity to the urethral meatus and external, colonized milieu.

**Figure 8:**
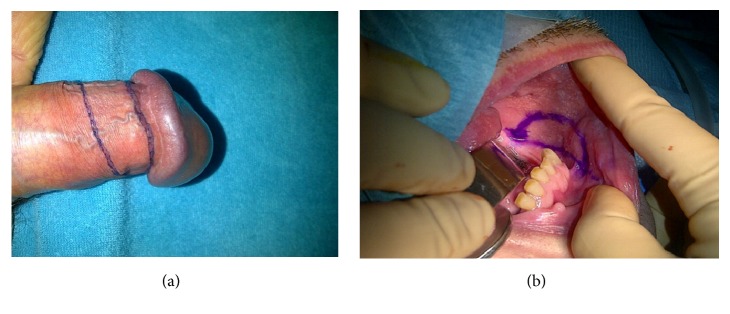
Preputial graft versus buccal mucosa graft.* (a) = preputial graft; (b) = buccal mucosa graft.*

*Preputial Grafts versus Buccal Mucosa Grafts. *Nowadays, the trend is to use buccal mucosa grafts over preputial grafts (Figure 8). However, this choice is mainly based upon expert opinion as convincing evidence to support this is currently lacking. Some retrospective reports have attempted to investigate this issue but could not justify to choose one over the other [65, 66]. Prospective, randomized, controlled trials will be necessary to truly justify this trend and to bring forward an evidence-based recommendation.

Despite its excellent success rates, buccal mucosa graft urethroplasty also brings along some drawbacks. In contrast to the use of preputial grafts, for instance, a second surgical site—the oral cavity—needs to be disinfected and prepared with sterile drapes, which lengthens the duration of the procedure. Also, the surgeon taking the oral graft needs to be familiar with the anatomy of the oral cavity. Furthermore, it cannot be denied that the created defect in the buccal mucosa may cause important pain and/or discomfort in these patients, possibly resulting in a longer hospital stay. Persisting oral symptoms may involve pain, swelling, numbness, diminished taste, speech problems, and/or an impairment of the mouth opening, smiling, or eating [67–69]. Nonetheless, the use of buccal mucosa grafts has been a major asset in the surgical repertoire to treat urethral stricture disease.

**Figure 9:**
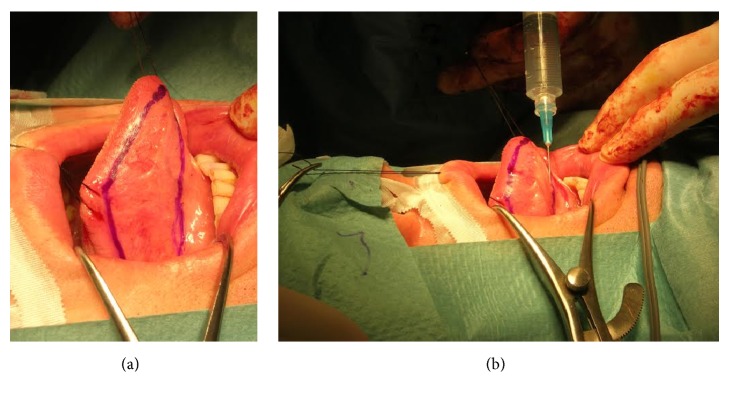
Lingual mucosa graft harvesting.* (a) = right, sublingual graft site; (b) = submucosal fluid injection to allow hydrodissection*.

*Lingual Mucosa Grafts. *Lingual mucosa grafts can be utilized as an alternative to buccal mucosa grafts (Figure 9). They are harvested from the sublingual region, respecting the sublingual nerve and the lingual papillae, which are important in the perception of taste. The main advantage of this graft site lies within its easy exposure, in contrast to an inner cheek. However, the graft length that can be obtained is limited to about 6.0-7.0 cm, depending on the size of the tongue. A randomized, controlled trial performed by Lumen et al. showed similar success and oral morbidity rates in buccal and lingual mucosa graft use, but the type of oral discomfort differed: lingual mucosa harvesting caused significantly more dysgeusia and problems with eating and speaking whereas buccal mucosa harvesting led to more oral tightness [70]. Be that as it may, the use of lingual mucosa grafts has also been an important asset in the armentarium of the reconstructive urologist.

**Figure 10:**
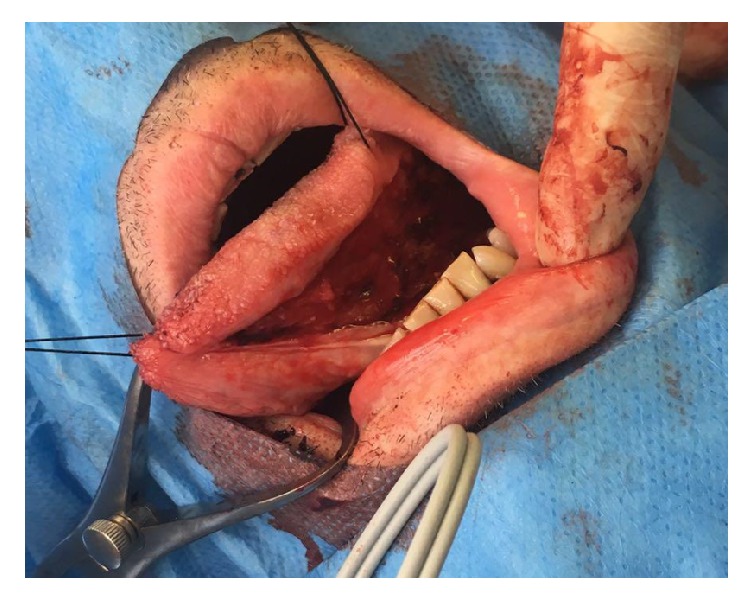
Nonclosure of the graft site.* The left, sublingual donor site is left open after thorough hemostasis*.

*To Close or Not to Close the Oral Graft Site. *A recent randomized, controlled trial by Soave et al. has shown that no closure of the donor site is noninferior to closure of the donor site regarding quality and intensity of oral pain (Figure 10) [69].

*Graft Placement. *Originally, free graft urethroplasty always involved a ventral placement of the graft on the longitudinally opened urethral stricture: the so-called “ventral onlay” free graft urethroplasty. Later, Barbagli et al. modified this technique and started placing grafts dorsally, against the cavernous bodies: the so-called “dorsal onlay” free graft urethroplasty [71]. This technique seemed to have the advantage of better graft fixation against its vascular bed and tended to cause less sacculation than ventral onlay procedures. A recent randomized, controlled trial, however, could not withhold any differences between both types of graft placement in the treatment of bulbar urethral strictures [72]. Hence, the choice between a ventral and a dorsal onlay urethroplasty is mainly left upon the surgeon's discretion, but it remains very case-specific and importantly driven by multiple patient and stricture characteristics (e.g., previous urethral surgery, stricture location, and quality of the local tissues). It is even possible to combine both ventral and dorsal graft placement in case of long-segment strictures or in case of very narrow strictures, as described by Hudak et al. [73] and Palminteri et al. [74].

Asopa has further modified this technique to a “dorsal inlay” free graft urethroplasty in which the urethra must not be dissected circumferentially to allow a dorsal onlay of the free graft [75]. In fact, this technique involves a ventral opening of the urethra followed by incising the dorsal urethral plate from the inside of the lumen. This adjusted approach has the advantage to be faster than a Barbagli procedure and can efficiently be administered when the surgeon intraoperatively finds that a ventral onlay procedure will not be possible. The success rates of a Barbagli procedure and an Asopa procedure are shown to be comparable [76].

It is a well-known fact that free graft urethroplasties at the penile urethra entail lower success rates than more proximal free graft procedures [64]. This observation may largely be explained by the relative paucity of corpus spongiosum at the more distal penile urethra. Hence, the graft almost fully depends on the vascular supply of the subcutaneous tissue in this region, which is strongly variable and much more tenuous than at the bulbar site. Against this background, it is assumed that dorsal graft placement is superior at the penile urethra, because then the cavernous bodies can act as a good vascular graft bed.

Later in the quest for minimally invasive urethroplasty, Kulkarni et al. described a new technique: the one-sided anterior urethroplasty with dorsolateral placement of the graft [44]. As such, only one side of the anterior urethra is dissected to allow graft augmentation while, on the other side, the vascular, neural, and muscular structures of the urethra are fully spared, which contributes to less tissue damage during urethroplasty. This may be particularly interesting at the penile urethra, where the vascular supply is more tenuous and rather vulnerable.

*Failure after Free Graft Urethroplasty. *The success of a free graft urethroplasty importantly depends on the relationship between the graft and its vascular bed. The graft has to be well in contact with a rich vascular bed in absence of any infection in order to survive. If one of these parameters is disturbed in any way, the risk of graft necrosis, and thus failure, exists. Even if none of these variables is disturbed, graft contracture can occur and may lead to restenosis of the urethra [77].

**Figure 11:**
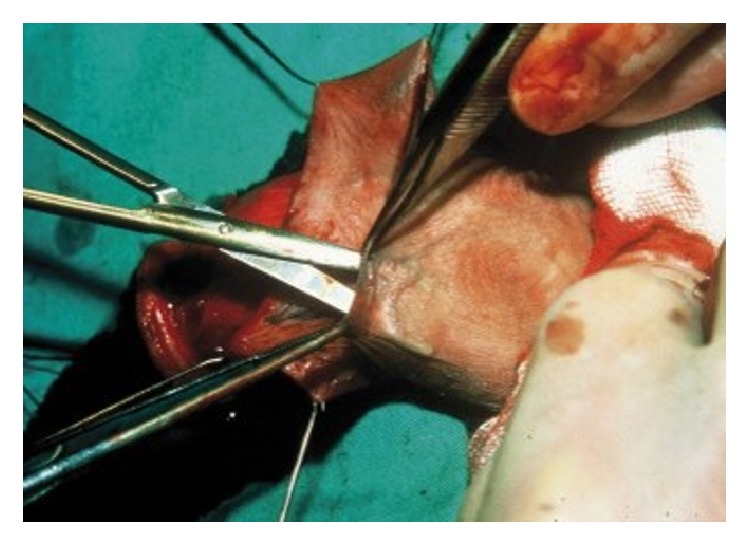
Duckett flap.* Mobilization of a pedicled Duckett flap (inner preputium)*.

**Figure 12:**
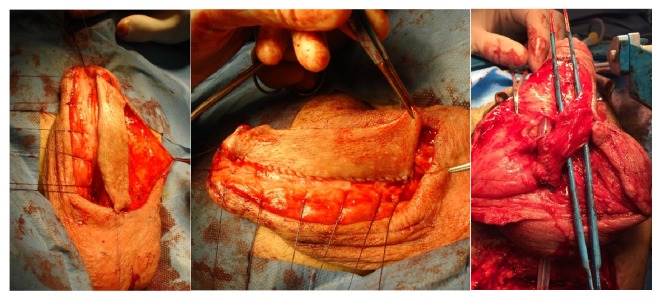
Orandi flap.* Mobilization of a pedicled Orandi flap (ventral longitudinal island; penile shaft skin)*.

*(3) Pedicled Flap Urethroplasty. *Pedicled flaps carry their own vascular supply in the pedicle and therefore can survive independent of the surrounding tissues. The preputium (Figure 11) and penile shaft skin (Figure 12) are both ideal sites to mobilize a pedicled flap from to augment the penile and/or the bulbar urethra up to the bulb of the corpus spongiosum. In complicated cases, scrotal flaps can also be administered, preferably after destruction of the hair follicles [78], and, exceptionally, even intestinal flaps may be used for extraordinary reconstructions [79].

Pedicled flap urethroplasty can be administered in basically every urethral stricture case, from the meatus up to the posterior urethra, and is associated with excellent success rates [64, 80–82]. Moreover, it is shown that tubularized flaps perform as well as patch procedures, in contrast to free graft procedures [80–82].

Undeniably, pedicled flap urethroplasty also brings along several postoperative side-effects, such as sacculation and intra-urethral hair growth, and should not be considered a first-choice treatment for relatively simple cases. However, though technically challenging, every urethral surgeon should master this variety of techniques, because, sooner or later, this will be the only pertinent option left.

*(4) Multistage Urethroplasty. *Given the outstanding success rates of one-stage urethroplasty, the indications for multistage procedures have diminished remarkably. Nowadays, staged interventions are mostly reserved for redo cases in which there's a complete lack of healthy tissue and only very precarious vascularization.

Almost every multistage urethroplasty technique is derived from the original Johanson technique [83]. The general principle of this technique is to first open the diseased urethra longitudinally and then suture the created urethral edges to the borders of penile/scrotal skin (depending on stricture location). As such, the diseased urethra is left open and a neo-meatus originates in a hypospadias position. This is considered the first stage of a Johanson procedure. The second stage of Johanson's procedure basically consists of retubularizing this marsupialized urethra around a transurethral catheter and is performed at earliest 3 months after the first stage. In some patients, however, the urethral plate will be of poor quality, even after several months. In these cases, it might be necessary to incise this fibrotic or ischemic plate dorsally and to augment the urethra with a free graft, placed against the corporal bodies [84–88].

After Johanson, several surgeons—Turner-Warwick [89], Gil-Vernet [90], and Blandy [91]—have further adapted the technique of staged urethroplasty, but the same idea is basically always respected.

*(5) Mesh Graft Urethroplasty or Multistage Oral Mucosa Graft Urethroplasty. *Mesh graft urethroplasty and multistage oral mucosa graft urethroplasty both represent a variation of multistage urethroplasty in which the disadvantages of tubularizing hairy skin segments (stone formation; infection) are avoided. With the mesh graft urethroplasty, a split thickness skin graft is harvested using a dermatome and meshed, and, with the multistage oral mucosa graft urethroplasty, a piece of oral mucosa is harvested and prepared as described above. The mesh graft or oral mucosa graft is then sutured in between the created urethral edges (after opening of the urethra) and the borders of the skin and, subsequently, during the second stage, there is no need to tubularize hair bearing skin, but only hairless graft material. These techniques are mostly reserved for complex reconstructions and mesh grafts are particularly interesting in cases with restricted graft/flap options [92, 93].

*(6) Definitive Perineostomy. *Nowadays, multistage urethroplasty is almost exclusively preferred in rather complex cases and in patients that have had numerous urethral interventions already. Often, these patients are perfectly happy to void without any problems after the first stage of an intended multistage procedure and wish to retain the created perineostomy without a second-stage procedure. This definitive perineal urethrostomy represents a well-accepted situation for many patients, especially multioperated and older ones.

**Figure 13:**
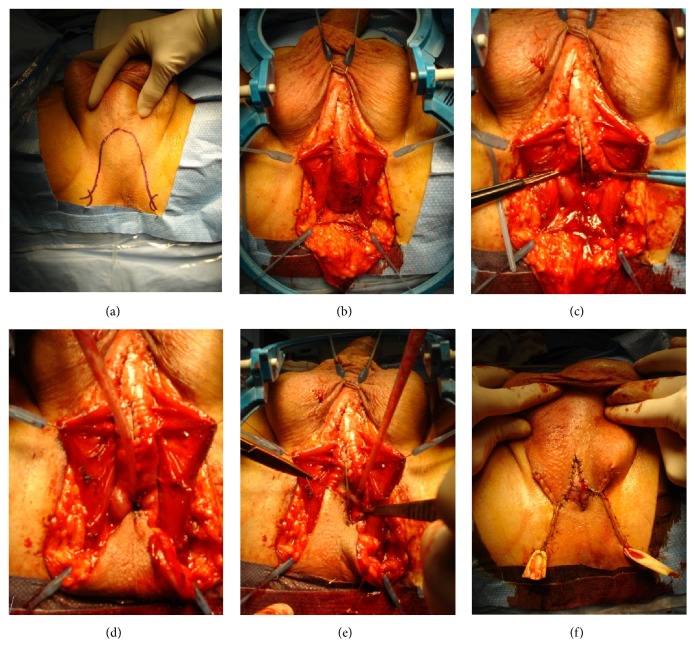
Blandy perineostomy.* (a) = inverted-U incision to create the Blandy flap; (b) = mobilized Blandy flap and exposure of the bulbar urethra; (c) = opening of the strictured area until patent proximal urethra is reached; (d) = suturing the tip of the Blandy flap to the deepest point of the opened urethra; (e) = further suturing the edges of the Blandy flap to the urethral edges; (f) = end-result of the Blandy perineostomy*.

For the creation of a definitive perineostomy, the Johanson and Blandy techniques are most frequently used and entail similar success rates [92]. With the Blandy technique, a perineal inverted-U incision is performed and the tip of this inverted-U flap is sutured against the deepest, most proximal part of the opened urethra (Figure 13). An alternative technique is the use of a 7-shaped flap, as described by French et al. [94]. In complex cases it might even be necessary to administer free grafts or mesh grafts in the creation of a perineostomy [95].

*(7) Tissue-Engineering in Urethroplasty. *Urethral reconstruction using matrix-bred tissue out of the patient's own urothelium or oral mucosa has recently been introduced as an alternative approach which mainly addresses the limitations inherent to the classic substitution urethroplasty. So far, tissue engineering with urothelium has been described in laboratory studies [96, 97] and, in 2018, Barbagli et al. have reported a success rate of 86% after MukoCell® graft urethroplasty in a clinical study (median stricture length of 5.0 cm and median follow-up of 55 months) [98]. In their technique, a 0.5 cm^2^ oral mucosa biopsy was harvested and sent to the laboratory for tissue engineering. After 3 weeks, the manufactured piece of tissue was sent back to the hospital and administered during urethroplasty (ventral onlay, dorsal onlay, dorsal inlay, and combinations).

Today, the largest limit of tissue engineering lies within its cost, but, definitely, this technique involves several advantages, especially when the classic substitution materials become scarce or even totally absent. Furthermore, it reduces the amount of donor tissue that is required for reconstruction and could therefore potentially reduce the side-effects of the graft site that are seen after a classic free graft urethroplasty [98]. Nonetheless, to date, there is no data to support this statement and future studies will be required to elucidate this issue. Also, the same conditions as in a classic free graft urethroplasty (close, immobile contact with a well vascularized graft bed in absence of infection) will be required to allow a successful procedure.

*(8) Combination of Techniques. *In case of multiple urethral strictures or very long, often complicated urethral strictures, a combination of the aforementioned techniques might be necessary to offer the patient a one-stage solution [99, 100]. In order to do so, a combined perineal and penile skin incision might be necessary. The most popular combination is probably represented by a free graft urethroplasty at the bulbar urethra, combined with a pedicled flap at the less vascularized penile urethra. However, basically every combination is possible but should be administered with common sense and respect to the urethral vascularization.

#### 5.3.5. Choice of Surgical Technique

For a primary urethroplasty, the surgeon should always opt for the simplest and most straightforward technique that yields the highest success rates. Ideally, the chosen technique also represents the treatment option that least compromises the therapeutic armamentarium that might be needed in the future as there is always a risk for urethroplasty failure requiring one or more salvage treatments [101].

For isolated, short (≤3.0 cm), bulbar urethral strictures, the authors follow the end-to-end urethroplasty recommendation of the ICUD, although a lot of controversy exists as mentioned above [46, 49, 50]. From the moment a stricture can no longer be treated with anastomotic repair, substitution urethroplasty will be required, in which a free graft urethroplasty represents the easiest and most evident technique to treat stricture disease from the meatus up to the posterior urethra [64]. Herein, penile urethral strictures are preferably augmented dorsally because of the rich vascular bed that is provided by the corporal bodies. In most cases, a free graft procedure will be favored over a pedicled flap procedure since flap urethroplasties are technically more demanding and interfere more with the external appearance of the genitals. Furthermore, free graft urethroplasties importantly rely on the quality of the urethral vascularization, which will be progressively impoverished, surgery after surgery. Hence, it makes sense to administer free grafts earlier in the treatment cascade, thus fully utilizing its window of opportunity.

The more redo procedures a patient has undergone, the harder it gets to choose between surgical approaches, as the options become sparser and gradually less favorable. In these cases, it is hard to stipulate general rules as these treatment decisions are very case-specific and need to be well deliberated. In long, complex and multioperated urethral strictures, a preputial or scrotal skin flap could remain a viable option, at least if this tissue is still available. An alternative is to perform a multistage urethroplasty or to construct a definitive perineostomy, which is often well-tolerated by patients with an elaborate history of urethral stricture treatments.

It should be underlined that this decision-making process may not be universal and that there is a lot of controversy about treatment algorithms for urethral reconstruction, especially in the bulbar urethra [46, 49, 50]. However, no matter which algorithm is used, it is of utmost importance that every surgeon eager to perform a urethroplasty masters the variety of techniques as described above, especially because an intraoperative shift from one technique to another is sometimes unavoidable. Hence, it must be stressed that urethroplasty should only be performed in recognized referral centers with sufficient volume.

#### 5.3.6. Peculiar Urethral Stricture Conditions and Locations

*(1) Posterior Urethral Strictures. *These strictures are characterized by the following:

- An anatomical proximity to the urethral sphincter and possible extension deeper than the urogenital diaphragm
- A distraction defect between the prostatic apex and the distal urethral portion
- Concomitant hematoma formation, infection, and/or previously failed urethral realignment procedures (endoscopic or open) which might lead to extensive stricture formation in the entire zone between both disrupted urethral ends, which makes it hard to recognize the local anatomy. Furthermore, in strictures related to a pelvic fracture, the displacement of bony fragments might hamper the exposure, which further complicates the surgical procedure
- In traumatic strictures, the possibility of coexisting damage to the penis, scrotum, and/or perineum, which may heavily compromise the available surgical options to restore the urethral continuity

The standard approach after trauma related urethral injuries involves the immediate placement of a suprapubic catheter [52] followed by a delayed urethroplasty, generally after 3 months, although, in reserved cases, a delay of 6 weeks may be enough [102]. Placement of the suprapubic tube should always be guided by imaging studies as the bladder may have been displaced in all possible directions depending on the impact of the trauma. Then, 3 months later, the ideal moment for an end-to-end urethroplasty is reached because the hematoma will have been resorbed and the distraction defect between both urethral ends will beat its minimum.

*(2) Meatal Strictures. *Meatal strictures may be located only at the meatus urethrae but can also expand in the navicular fossa or the entire transglandular segment. In some cases, the meatal stricture is part of an entire diseased anterior urethra.

A meatal stricture is often considered as a minor and rather benign condition, although, in every case, the clinician has to consider the possibility of underlying lichen sclerosus. Furthermore, obtaining a perfectly functional and esthetic result is harder than it may seem. Herein, the severe deviation of the urinary stream represents the most bothersome complication as it will obligate the patient to void in a sitting position.

The true extent of a meatal stricture is often difficult to estimate preoperatively. In pronounced strictures, a RUG will usually be impossible to perform and, during a VCUG, the entire proximal urethral segment will dilate but will only poorly reveal the true length of the stricture. In fact, the most reliable length measurement takes place in the operating theatre. Hence, the surgeon starting the meatoplasty should always master a variety of techniques as every patient will need an individualized surgical approach.

**Figure 14:**
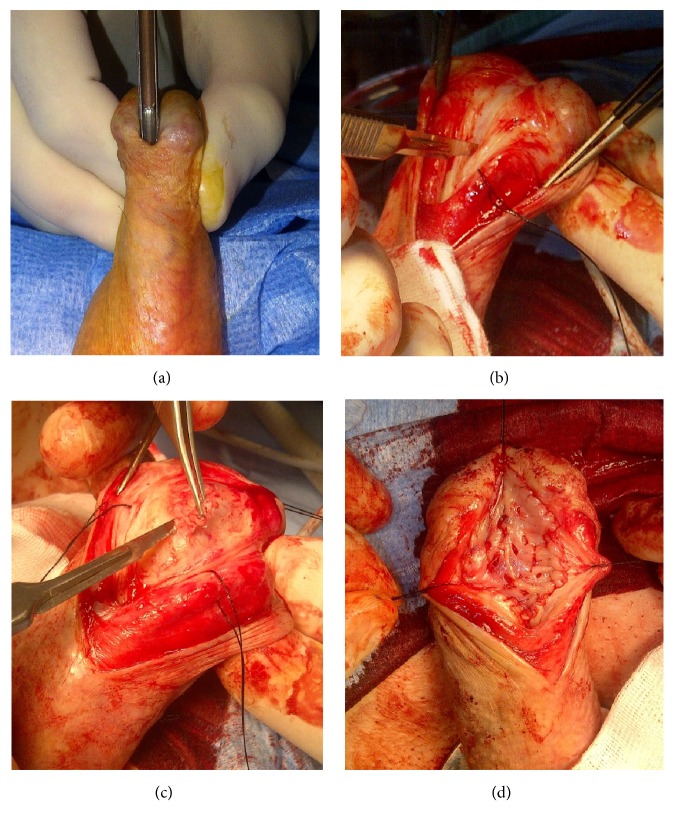
Asopa technique for meatal and navicular strictures.* (a) = opening of the meatal stricture with a grooved director; (b) = incision of the dorsal urethral plate; (c) = excision of fibrotic tissue; (d) = dorsal inlay of buccal mucosa graft*.

Several techniques have been described to reconstruct the urethral meatus [103]. Many of these techniques, however, do not involve a closure of the glans and thus leave behind a hypospadias neo-meatus. From both a functional and an esthetic point of view, one should always attempt to close the glans and to restore the distal penile anatomy as meticulously as possible. Penile skin flap meatoplasty represents one of the suggested techniques but is nowadays gradually abandoned as it often leads to a slightly hypospadias meatal position afterwards. During this technique, a small pedicled penile skin flap can easily be mobilized after a subglandular incision to access the meatal urethra. An alternative approach is to cleave the glans and to dorsally incise the urethral plate into healthy, well vascularized tissue. Thereafter, an according graft can be harvested and dorsally laid in, according to Asopa [75], and the glans wings can be closed in two firm layers to prevent fistulas and glandular dehiscence (Figure 14). More recently, another alternative for meatal reconstruction has been described by Nikolavsky and involves transmeatal buccal mucosa graft repair of the meatus and navicular fossa [104]. During this procedure a wedge of scar tissue is cut out ventrally through the meatus and the tip of the buccal mucosa graft is put into the apex of the created defect using a double-armed suture following the inside-out principle. Thereafter, both sutures are tied externally and the same principle is used for the edges of the graft until adequate fixation is obtained.

The use of genital skin must be avoided in all patients with a lichen sclerosus related stricture as it can lead to a failed procedure [9]; in these cases, oral mucosa must be administered [105].

*(3) Lichen Sclerosus Related Strictures. *Lichen sclerosus is a chronic, inflammatory skin condition with a specific predilection for the genital region. Furthermore, this disease may importantly affect the penile as well as the bulbar urethra [8, 9]. These strictures are associated with higher failure rates after urethroplasty than strictures of any other etiology and require a specific therapeutic approach [9].

As mentioned above, it is generally accepted that lichen sclerosus related strictures are not to be treated with skin as substitution material, but with oral grafts [105]. Nonetheless, it remains unclear whether oral mucosa grafts are actually resistant to this disease or not.

Based on good dermatological results after high-dosed, locally applied corticosteroids, its value has also been evaluated for intra-urethral administration and has shown satisfying results in small patient series [106, 107]. It makes sense that these substances would also be beneficial as an adjuvant to urethroplasty, but so far there is no evidence to support this statement.

*(4) Urethral Strictures after Local Prostate Cancer Treatment. *All local treatment options for prostate cancer (radical prostatectomy, external radiation therapy, brachytherapy, and focal prostate cancer treatments like high intensity focused ultrasound (HIFU) or cryotherapy) may contribute to subsequent stricture formation, located between the bladder neck and bulbar urethra [108]. These strictures are usually characterized by intense fibrosis and poorly vascularized surrounding tissues which may hamper a successful and uncomplicated surgical procedure. In most of the cases, an end-to-end urethroplasty is the technique of choice, especially because these urethral strictures are usually rather short [109–112]. The authors believe that if one of both urethral ends is well vascularized, a decent to good success rate may be expected [111]. Substitution urethroplasties have also been described for this indication and seem to bring along a successful surgical outcome [109, 112, 113]. However, these numbers are based on highly selected patient series and the true success of these procedures may have been overestimated.

It must be acknowledged that, overall, these patients are hard to treat and that urethroplasty in these cases holds an important risk for failure. Furthermore, the presence of a stricture may have concealed a problem of underlying incontinence, which may suddenly appear after a successful procedure in which the urethral patency has been restored. Hence, all patients should be thoroughly informed preoperatively and well counseled about the potential consequences of treating their stricture.

#### 5.3.7. Impact of Urethroplasty on Sexual Life

In a literature review incorporating 36 studies with a total of 2323 patients, persisting de novo erectile dysfunction has been described in 1% of the patients after urethroplasty [114]. This number strongly varied between different studies and ranged from 0% to 38%, which may be attributed to differences in patient and stricture characteristics, types of repair, definitions of erectile dysfunction, and methods of questioning. On the other hand, a transitional decline in erectile function shortly postoperative (6 weeks) has been described with spontaneous resolution after 6 to 12 months [115].

With the classic transecting end-to-end urethroplasty, the erectile tissue is directly damaged during surgery and thus one could expect that erectile function importantly decreases after this procedure. This assumption has been illustrated by a study of Ekerhult et al., although they could only withhold a 5% incidence rate of de novo erectile dysfunction after end-to-end procedures [116]. Herein, the question remains whether nontransecting end-to-end urethroplasty is linked to lower postoperative sexual dysfunction without impeding the surgical success rates of urethroplasty by excision and primary anastomosis [59]. As regards free graft urethroplasty with buccal mucosa, studies have shown that these procedures do not impact the patients' postoperative erectile function [117, 118].

Ejaculatory function is often better after urethroplasty than before, provided the use of a technique in which the continuity of the bulbospongiosus muscle is actively restored during the multilayered closure of the perineum [119, 120].

#### 5.3.8. Postoperative Course

After urethral reconstruction, it is important to provide urinary derivation as urine extravasation at the recently operated region might lead to important complications, such as abscess formation and phlegmon. When a free graft has been used, the authors routinely leave a 20 Fr transurethral catheter in place to avoid prolapse of the graft into the urethral lumen and to allow close contact between the graft and its vascular bed. In other cases, urinary derivation is assured through a 16 Fr transurethral catheter or a suprapubic tube. Nonetheless, it should be underlined that catheter use after urethral reconstruction is extremely variable between different urethroplasty centers, without clear data if one regimen is truly better than the other.

Most patients are discharged from the hospital on the second or third postoperative day with the indwelling catheter in place. At that moment, instructions for wound care are provided, which are specifically important in patients with a perineal wound. These wounds need to be kept dry and clean. Therefore, the use of a hair dryer (3 to 4 times a day) and repeated disinfection is advised, a method that was copied from wound care principles after episiotomy in females.

Routine perpetuation of the antibiotic treatment regimen must be limited to those patients in which a preoperative urinary tract infection has been established. In these cases, appropriate antibiotics (according to the antibiogram) are continued for a maximum of 5 days since any longer use will only contribute to the problem of resistant microorganisms. There is no clear evidence to support this advice in urethroplasty, although the authors base this recommendation upon the general rules and principles of antibiotic therapy.

After 7 to 14 days, the first postoperative visit is scheduled and involves the execution of a VCUG, after filling the bladder with contrast medium through the indwelling catheter. Some authors argue the value of a routine postoperative urethrography as only few patients will show contrast extravasation requiring a catheter replacement [121, 122]. However, these authors routinely leave the catheter for 3 weeks instead of 1 or 2 weeks. It has been established that indwelling catheters bring along important side-effects and complications and thus it might certainly benefit the patient to remove the catheter as early as possible [123]. Moreover, it has been demonstrated in our department that catheter removal after 8 days is as safe as prolonged catheter dwell-time and that extravasation at first VCUG has an important negative prognostic value [124].

#### 5.3.9. Follow-Up after Urethroplasty

To date, the ideal follow-up of patients after urethroplasty remains poorly defined. At Ghent University Hospital, patients are scheduled to revisit after 3 months, after 12 months, and annually thereafter. During these visits, anamnesis, physical examination, uroflowmetry, and Urethral Stricture Surgery Patient Reported Outcome Measures (USS-PROM) questionnaires are routinely administered [125, 126]. Additional urethrography and/or cystoscopy are/is only performed in case of obstructive voiding symptoms or a maximal flow rate of < 15 mL/s.

To date, there is no clear consensus about standard administration of urethrography and/or urethroscopy during follow-up. There is, however, a remarkable trend to use patient-reported outcome measures (PROMs) after urethral reconstruction. In 2011, Jackson et al. created and validated the USS-PROM, a questionnaire specifically made for patients after urethroplasty [125]. Later, numerous validated translations have been reported and implemented in routine clinical practice [125–132].

The optimal follow-up schedule will need further elucidation in the future. Presumably, this will not be a story of “one-size-fits-all” as urethral stricture disease entails a very heterogeneous patient cohort which certainly demands patient-adapted follow-up strategies.

## 6. Conclusion

Male urethral stricture disease embodies a very heterogeneous condition in which thorough knowledge about anatomy, etiology, symptoms, diagnosis, and treatment aspects is crucial in optimizing care of these patients. Future prospective research will be warranted to gain further evidence and to refine the current practice of managing male urethral stricture disease.

## Abbreviations

CT: Computed tomography
DVIU: Direct vision internal urethrotomy
EPA: Excision and primary anastomosis
HIFU: High intensity focused ultrasound
ICUD: International Consultation on Urologic Diseases
MMC: Mitomycin C
MRI: Magnetic resonance imaging
Qmax: Maximal urinary flow rate
RR: Relative risk
RUG: Retrograde urethrography
USS-PROM: Urethral Stricture Surgery Patient Reported Outcome Measures
VCUG: Voiding cystourethrography.
